# Challenges of vaccination and herd immunity in COVID‐19 and management strategies

**DOI:** 10.1111/crj.13543

**Published:** 2022-09-29

**Authors:** Jun She, Dongni Hou, Cuicui Chen, Jing Bi, Yuanlin Song

**Affiliations:** ^1^ Department of Pulmonary and Critical Care Medicine, Zhongshan Hospital Fudan University Shanghai China

**Keywords:** COVID‐19, herd immunity, prevention, SARS‐CoV‐2, vaccination

## Abstract

Coronavirus disease 2019 (COVID‐19), the highly contagious viral disease caused by severe acute respiratory syndrome coronavirus 2 (SARS‐CoV‐2), has spread worldwide with millions of cases and more than 5 million deaths to date. SARS‐CoV‐2 has caused serious damage all over the world with many countries experiencing the third or the fourth wave of the viral disease outbreaks, mainly due to the emergence of mutant variants. Those who unvaccinated remain most vulnerable to COVID‐19 and its variants. COVID‐19 vaccination, along with prevention strategies, is a critical measure to defense against the disease. COVID‐19 vaccination can reduce the spread of virus and help protect susceptible population. Although herd immunity might not be realized solely by vaccination, COVID‐19 vaccines have been proved to be effective in reducing the risk of severe disease, hospitalization, and even death. It is recommended that people get vaccinated as soon as they are eligible. This review summarizes the recent SARS‐CoV‐2 variants that brought challenges for vaccination and herd immunity and discusses promising management strategies.

## Graphical abstract

A. Vaccination induces production of protective antibodies to block the receptor binding domain (RBD) of spike protein from binding to the host angiotensin‐converting enzyme‐2 (ACE‐2) receptor; B. Mass vaccination in population establishes immune barrier, while no vaccination or few vaccination is at risk of COVID‐19 spreading.

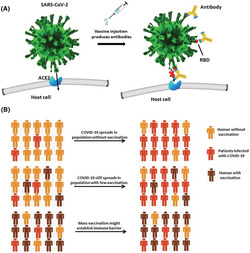

**List of Abbreviations**
ACE2: angiotensin‐converting enzyme 2
ARDS: acute respiratory distress syndrome
COVID‐19: coronavirus disease 2019
IPC: infection prevention and control
PHSM: public health and social measures
RCTs: randomized controlled trials
SARS‐CoV‐2: severe acute respiratory syndrome coronavirus 2
VOC: variants of concern
VOI: Variants of Interest
VUM: variants under monitoring
WHO: world health organization

## 1 BACKGROUND

Coronavirus disease 2019 (COVID‐19), the highly contagious viral disease caused by severe acute respiratory syndrome coronavirus 2 (SARS‐CoV‐2), has spread worldwide with experiencing many waves of the viral disease outbreaks,^1^, ^2^, ^3^, ^4^ mainly due to the emergence of mutant variants.^5^ Limiting the continuing spread of the virus and its variants has become a great challenge. This review is a comprehensive overview of different variants of SARS‐CoV‐2 and the challenges in vaccination and herd immunity.

## 2 SARS‐COV‐2 VARIANTS

Viruses, like SARS‐CoV‐2,^6^ continually adapt to their new hosts. They are prone to develop new genetic mutations over time, resulting in mutant variants that may have characteristics different from its ancestral strains. The receptor binging domain (RBD) in spike protein of SARS‐CoV‐2 binds to the host angiotensin‐converting enzyme‐2 (ACE‐2) receptor with high affinity, which is the origin of most clinically concerned SARS‐CoV‐2 variants.^7^ Most of the mutations have little to no impact on the virus' properties, while a few mutations may alter its pathogenicity and increase spread ability, disease severity, or decrease the protection from vaccines and medicines.^5^ For example, the alpha variant found in the UK has 10 mutations in spike protein; Delta variant has some mutations in S1 subunit, including three mutations in RBD. These mutations not only improve the affinity between RBD and ACE‐2 but also increase its ability to escape the host immune system. The junction structure of the spike protein would be modified by the TMPRSS2 enzyme of host when the spike protein was combined with ACE‐2. After the spike protein cut by TMPRSS2 enzyme, its hydrophobic amino acids were exposed to fuse with the host cell membrane.^8^

Continuous evolution of SARS‐CoV‐2 mutant variants has brought challenges to global public health. The world health organization (WHO) have been monitoring and assessing the evolution of SARS‐CoV‐2 since January 2020. The emerging variants were classified as the variants of interest (VOI) or variants of concern (VOC) based on their attributes and prevalence (Table 1). If significant amino acid substitutions were identified, the national authorities and the public health institutions will be informed about any changes that needed to respond to the variants and prevent its spread. The WHO was also listed six variants as variants under monitoring (VUM).^9^

**TABLE 1:** There were the main of four VOCs and two VOIs in SARS‐CoV‐2 variants have been identified

	Label	Pango lineage	Earliest samples	Date of designation
VOC	Alpha	B.1.1.7	United Kingdom Sep 2020	Dec 2020
	Beta	B.1.351	South Africa May 2020	Dec 2020
	Gamma	P.1	Brazil Sep 2020	Jan 2021
	Delta	B.1.617.2	India Nov 2020	May 2021
	Omicron	B.1.1.529	Multiple countries Nov 2021	Nov 2021
VOI	Lambda	C.37	Peru Dec 2020	Jun 2021
	Mu	B.1.621	Colombia Jan 2021	Aug 2021

Abbreviations: VOI, variants of interest; VOC, variants of concern.

Globally, 193 countries, territories, or areas have been reported cases of the alpha variant, while 142 countries reported cases of beta variant, and 96 countries reported cases of gamma variant. For the delta variant, it has been reported in over 185 countries so far.^10^ Active and immediate action is needed to understand potential impacts of the VOCs on characteristics of COVID‐19, including epidemiology, severity, effectiveness of public health and social measures, diagnostic methods, immune responses, and antibody neutralizing ability.

SARS‐CoV‐2 alpha variant was highly transmissible.^11^ The alpha variant included 17 mutations in viral genome. Among them, eight mutations including Δ69‐70 del, Δ144 del, N501Y, A570D, P681H, T716I, S982A, and D1118H are in the spike protein. N501Y increased affinity of the spike protein to ACE‐2 receptors and enhanced the ability of virus attachment and subsequent entry into host cells.^12^, ^13^ Cases infected with alpha variant had severe symptoms, such as continuous cough and a large amount of mucus with virus flowing out of mouth and nose.

After alpha, beta variant acquired higher infectiousness by immune escape. The infection rate of beta variant was about 50% higher than that of the original SARS‐CoV‐2 virus, and the risk of hospitalization, severity, and mortality were also higher. Beta variant also increased its transmission by N501Y mutation and avoided the human immune system tracking by E484K mutation.^14^

Gamma variant was the biggest threat of epidemic in South America. Patients with the infected variant might have reinfection. There was two times of transmissibility in Gamma variant than the original SARS‐CoV‐2 virus.^15^

Delta variant was responsible for the deadly second wave of COVID‐19 in India.^9^ Delta variant harbor 10 mutations in the spike protein^5^ and caused more infections and spread faster than alpha and beta. It might cause more severe illness than previous strains in unvaccinated people.^16^ In addition, the risk of hospitalization in delta variant related cases was higher, approximately twice that of alpha variant. In addition, delta variant had enhanced infectivity and stability of viral replication in human lung epithelial cells and primary human airway tissues due to D614G mutation, supporting clinical findings of enhanced viral loading in the upper respiratory tract and increased transmissibility.^17^

Omicron variant has been considered to be the most ferocious new variant by far. There were 32 mutations in omicron variant, which is twice that of delta variant, and the number of spike protein mutations is the largest. Ten mutants were found in the RBD of omicron variant and only two in delta variants. These mutations in spike proteins had been found in delta and alpha variant and were relate to transmissibility or immunity evasion.^18^ Those unvaccinated were the most vulnerable to omicron variant.^19^ And Altarawneh et al.'s study showed that the protection of a previous infection with SARS‐CoV‐2 against BA.4/BA.5 was lower than that against BA.1/BA.2 consistent with BA.4/BA.5's greater capacity for immune system evasion than that of BA.1/BA.2.^20^ Epidemiological characteristics, transmissibility, and immune escape of omicron variant remain to be comprehensively identified in the future.

## 3 VACCINATION

Vaccination against COVID‐19 has become a critical public health solution as global pandemic of COVID‐19 continues to worsen throughout the world. There were more than 400 randomized controlled trials (RCTs) and 200 nonrandomized studies of vaccines for COVID‐19.^21^ Although vaccination for prevention of COVID‐19 is developing at an unprecedented rate, recent data showed that the decreased protection may be due both immunity wane over time^22^ and the highly contagious COVID‐19 variants which threatened to overturn the vaccination efforts that has been made.

Many studies showed effectiveness of vaccines in COVID‐19. Vaccines reduced the risk of symptomatic and asymptomatic disease and might therefore reduce the spread of SARS‐CoV‐2.^22^ However, SARS‐CoV‐2 mutant variants not only had enhanced transmissibility but also effectively avoided human immune response. For instance, alpha variant with spike protein mutation 69‐70del may escape from host immune system^23^, ^24^ In addition, the antigenic drift occurred in accumulation of mutations in SARS‐CoV‐2 might be nightmare. Antigen drift is a classical phenomenon in virology. It refers to the small variations in virus antigens caused by individual mutation of virus genome in natural epidemic. The variation is different from virus recombination and will not have a large‐scale impact on virus epidemic. However, if antigen drift continued to accumulate and the rate of accumulation continued to increase for a long time,^25^ people will need revaccinate to acquire protection against new variants, such as in the scenario of seasonal influenza.

Some vaccines could still provide fine protection for SARS‐CoV‐2 variants. In the aspect of vaccine efficacy, AstraZeneca vaccine was effective against alpha variant with an effectiveness of up to 90% after a second regimen.^26^ It was reported that the effectiveness of Pfizer‐BioNTech vaccine to beta variant was 91% in Qatar.^27^ Although beta variant had the ability of immune escape, the vaccine remained effective, especially for the severe cases. The effectiveness of Johnson & Johnson vaccine against gamma variant was 66% in a Brazilian trial,^28^ but distribution of the vaccine was halted due to its potential side effects of causing rare blood clots. Pfizer‐BioNTech vaccine (BNT162b2) and AstraZeneca vaccine (ChAdOx1) were effective for Delta variant. The effective rate of Pfizer‐BioNTech vaccine against delta variant was 88% after two doses of vaccination and 36% after one dose, while 67% after two doses and 30% after one dose for AstraZeneca vaccine. The difference in effectiveness of AstraZeneca vaccine between alpha and delta variants was small (74.5% vs. 67.0%).^29^ More vaccine researches for delta variants are still ongoing.

Emergency use of a single booster shot of the Pfizer‐BioNTech vaccine has been authorized in the United States. Certain populations were eligible to receive a booster shot at least 6 months after receiving their second shot, including people aged ≥65 years, people aged ≥18 years who have underlying medical conditions, and people aged ≥18 years who live or work in high‐risk settings.^22^

## 4 HERD IMMUNITY

Herd immunity is an important concept for epidemic control. A proportion of population needs to be immuned by natural infection or vaccination to stop generating large outbreaks.^30^ It is reported that the protection of previous pre‐Omicron infection against Omicron BA.4/BA.5 reinfection was 15.1%–28.3%, while the protection of previous Omicron infection involved the Omicron BA.1 or BA.2 against Omicron BA.4/BA.5 reinfection was 76.1%–79.7%.^20^ Vaccines have created a window of hope for more effective fight against COVID‐19 pandemic by achieving herd immunity. Most countries have initiated vaccination programs to control transmission and decrease disease burden.^31^ Despite the challenges in managing COVID‐19 pandemic waves in different contexts and capacities, several COVID‐19 vaccines have been widely used with over 7 billion doses as of Nov 2021.^1^

However, the impacts of the pandemic are felt unequally across the world, with varying public health strategies leading to different epidemic trajectories. Due to the challenges of vaccine supply and promotion, coupled with the potential emergence of highly infectious SARS‐CoV‐2 variants, it was controversial whether current vaccination strategies would generate herd immunity. A study from Korea showed that even with the SARS‐CoV‐2 variants, vaccination program may significantly reduce the disease burden associated with COVID‐19, such as symptomatic infection, hospitalization, and death.^32^ However, with the emergence of the delta variant, it became harder to achieve herd immunity as previously estimated.

The issues in dealing with COVID‐19 pandemic were how and when herd immunity would be achieved and at what we should cost, especially as more transmissible SARS‐CoV‐2 variants continue to emerge.^30^ Some data‐driven models of SARS‐CoV‐2 transmission showed that vaccine‐induced herd immunity would require coverage of 93% or higher, if the vaccine efficacy against infection was 74%. Herd immunity for new variants, such as alpha or delta, may require more efficacious vaccines and coverage above 80%–90%.^33^ There were 67.1% people reported to be willing to accept vaccination, while 9.0% refused it. About 35.5% people reported vaccine hesitancy, including 48.8% acceptors with doubts, 39.4% refusers, and 11.8% delayers.^34^ The current coverage was pretty low, far from reaching the requirements of herd immunity and insufficient to stop the pandemic. More flexible and comprehensive efforts should be taken to improve people's confidence and willingness for vaccination and to develop more efficacious vaccines against SARS‐CoV‐2 variants.^35^ Since herd immunity cannot be achieved what should be done?

## 5 MANAGEMENT STRATEGIES

In transmission of SARS‐CoV‐2 variants, public health and social measures (PHSM) including infection prevention and control (IPC) measures have been proved effective in reducing COVID‐19 cases, hospitalization, and death. COVID‐19 vaccination can also reduce the spread of SARS‐CoV‐2 and help to protect both oneself and people around. Many countries struggling with continuous waves of COVID‐19 are encouraged to strengthen existing PHSM and IPC measures and monitor the spread of SARS‐CoV‐2 variants by detecting unusual epidemiological events. Reducing transmission and avoiding introductions of animal populations through established and proven disease control measures are essence of the global strategies to reduce occurrence of mutations.^36^

## 6 PRIORITIZATION POPULATION

The prioritization population has been recommended for vaccination based on underlying physiological differences and epidemiological features of COVID‐19 deaths during the first wave.^37^ Complications of severe COVID‐19 such as acute respiratory distress syndrome (ARDS) and death were observed in individuals with co‐morbidities, for example, obesity, arterial hypertension, or chronic kidney disease and in the elderly. Thus, the priority population includes those with health conditions that were predispose to severe morbidity from infections of COVID‐19 and the elder, especially those in care homes. The front‐line health‐care staff and those working in essential services were also priority population.

## 7 CHILDREN

The incidence and mortality of COVID‐19 were lower in children than adults in the early waves of infection. And children infected with SARS‐CoV‐2 present mild symptoms or were asymptomatic. However, the mortality of COVID‐19 was higher in children (aged 0–18 years) during recent delta variant outbreak in Malaysia.^38^ The surged data of cases among children have also been recorded in the United States and England. Moreover, COVID‐19‐associated hospitalization rates were nearly fivefold among children and adolescents, especially in the unvaccinated adolescents, rate of whom was about 10 times higher compared with the vaccinated.^39^ The prevent and control strategies in some countries have authorized to use COVID‐19 vaccine for children ages from 5 to 11 years to reduce transmission and possible severe outcomes.^40^

A number of population‐based SARS‐CoV‐2 seroprevalence and viral shedding studies investigated whether the infection rate in children was the same as that in adults. Children infected with SARS‐CoV‐2 spread the virus in their respiratory tract and possible in their feces.^41^ Among individuals who detected SARS‐CoV‐2 positive at the same time point after the onset of symptoms, RNA loads of SARS‐CoV‐2 virus in the respiratory track were similar in children and adults.^42^ Some vaccines such as CoronaVac, Pfizer‐BioNTech (BNT162b2), and mRNA‐1273 have been evaluated for safety, tolerability, and immunogenicity in children and/or adolescents, which proved vaccine efficacy in reducing symptoms, hospitalization, and death.^43^, ^44^, ^45^ The side effects of mRNA vaccines such as myocarditis should be monitored.^45^ Online education at home, instead of education at school, maybe recommended measures for children and adolescents currently.

## 8 IMMUNOCOMPROMISED PERSON

Immunocompromised patients including those with solid tumors or hematologic malignancies, solid organ transplant recipients, hematopoietic cell transplant recipients and patients with human immunodeficiency virus (HIV), and primary immunodeficiency were at higher risk of developing COVID‐19‐related severe outcomes.^46^ The population needs special attention, as infections are among the most common causes of mortality in them, although the data from the COVID‐19 rheumatology registry so far have been reassuring and has not revealed an increased risk of COVID‐19 complications in immunocompromised patients except those on moderate or high doses of corticosteroids.^47^ A reduced immune response was observed after two doses of COVID‐19 vaccine in patients with solid and hematological cancer and in transplant recipients.^48^ Planning the vaccination of the immunocompromised patients to ensure maximum possible seroprotection will be needed.

## 9 ALLERGIC PERSON

To obtain safe and effective outcomes, applying risk–benefit assessments and shared decision models to the massive COVID‐19 vaccination is needed. The potential life‐saving benefit of vaccination in the setting of global pandemic makes it important to assess every patient who may have an allergic reaction to prevent unnecessarily refusal of vaccination. For highly allergic individuals, professional guidance recommended appropriate information and support to receive the COVID‐19 vaccines. The patients with potential allergic reaction to the first dose of SARS‐CoV‐2 vaccine should be carefully evaluated to determine whether there is a real allergic reaction occurred and decide whether and how to receive the second dose.^49^

## 10 VACCINATION DECISION‐MAKING

Lessons learned from other outbreaks, for example, Ebola, suggested that there are individual differences in vaccine confidence gap.^50^ Some people may hesitate vaccination because of beliefs that they have a low risk of infection. Some may be worried about the safety of vaccines, while others may be hesitated due to a lack of trust in the health system. Understanding their different motivations could be a good entry for developing strategies to deal with obstacles.

Vaccination decisions are influenced by people's social interactions, such as family members, friends, health professionals, and others who interact with them. The encouragement and social pressure from people whom they respect and trust increased vaccine uptake. The willingness or unwillingness to vaccinate can be transmitted through the social cascade when one group affects another group, and then the two influence the third, and so on. Targeting health professionals who have more opportunities to influence vaccination behavior would have a greater impact on behavior changes.^51^

Trust in COVID‐19 vaccine would be crucial to its success. Further trials to investigate efficacy in older adults are now needed. Efficacy is considered including the longevity effects, reduction in severe infections, and safety in high‐risk groups. The approaches to enhance vaccine acceptance among people were important for the mass vaccination: (1) highlighting norms in favor of vaccination, (2) leveraging the role of health professionals, (3) supporting health professionals to promote vaccination, and (4) amplifying endorsements from trusted community members.^52^

## 11 CANDIDATE THERAPEUTIC DRUGS

A novelty of COVID‐19 oral antiviral candidate PF‐07321332 (Paxlovid).^53^ has been tested in a randomized, double‐blind phase II/III study by Pfizer among nonhospitalized adult patients with COVID‐19, who were at high risk of progressing to severe illness. The interim analysis showed reduced risk of hospitalization or death by 89% compared with placebo in patients treated within 3 days of symptom onset. The original report is “0.8% of patients who received PAXLOVID™ were hospitalized through Day 28 following randomization (3/389 hospitalized with no deaths), compared with 7.0% of patients who received placebo and were hospitalized or died (27/385 hospitalized with seven subsequent deaths)”.^54^ It was better than another COVID‐19 oral antiviral candidate Molnupiravir by Merck and Ridgeback, which reduced the risk of hospitalization or death by about 50%. These data were also far better than Oseltamirvir—a neuraminidase inhibitor which reduced the risk of death by 19% compared with no treatment in influenza A.^55^

PF‐07321332 was designed to block the activity of the SARS‐CoV‐2‐3 CL protease, an enzyme that is required in replication. PF‐07321332 inhibited viral replication at a stage known as proteolysis, which occurs before viral RNA replication, while it did not demonstrate evidence of mutagenic DNA interactions in preclinical studies.^53^ Combination with low‐dose of ritonavir might slow its metabolism or breakdown to maintain its activity and concentration for longer periods. PF‐07321332 has also been demonstrated potent antiviral activity against other known coronaviruses, suggesting its potential as a therapeutic for multiple types of coronavirus infections.

## 12 UNIVERSAL VACCINES

Similar to influenza virus, vaccines might be formulated every year to match the SARS‐CoV‐2 variants, as they evolve antigenically owing to antigenic drift.^56^ If the vaccine and variants were mismatched, the efficacy of vaccine would be low. Increased attention and monitoring antigenic drift could further increase the antigenic match between vaccines and variants. Furthermore, antigenic drift of T cell epitopes has been observed but not as frequently as antibody‐mediated drift.^57^ The viral spike protein was identified as the main vaccine target as it contains the RBD allowing host cell entry. Neutralizing antibodies against the spike protein have been described in SARS‐CoV‐2 vaccination, while the emergence of antibody‐resistant SARS‐CoV‐2 variants might limit the therapeutic usefulness of monoclonal antibodies. In addition, recent studies demonstrated that the emerged SARS‐CoV‐2 mutant D614G in patients' sera suggested the cross‐protection from reinfection by strain. However, patients' SARS‐CoV‐2 sera commonly lacked the cross‐neutralization of homologous bat coronavirus (WIV1‐CoV), which has not yet crossed the species barrier.^58^ Targeting the RBD, identifying epitopes and features, and developing universal vaccines may provide protection against antigenically distant coronavirus to prevent future COVID‐19 pandemics.^58^, ^59^

## 13 POST‐COVID‐19 AND PREVENTION

As living together with COVID‐19, millions of people have multiple episodes of a mild sore throat and lost productivity in few days with the highly transmissible and unstable virus. Post‐COVID‐19 syndrome is one of the challenges, having become increasingly common as the pandemic evolves. The latest estimates suggest that 10% to 20% of the COVID‐19 patients who undergo an acute symptomatic phase are experiencing effects of the disease beyond 12 weeks after diagnosis.^60^ Breathlessness, fatigue or muscle weakness, sleep difficulties, persistence of smell, and taste disturbances and neuropsychological symptoms were the most frequently reported. What is more, patients who were severely ill during hospital stay had serious impaired pulmonary diffusion capacities and abnormal chest imaging manifestations and are the main target population for intervention of long‐term recovery.^61^ It was reported that exercise has been shown beneficial in both patients' symptoms and its possible pathogenic mechanisms. It is worth considering that the potential favorable effect would bring the recovery of post‐COVID‐19 patients.^60^, ^62^ A new clinical study is ongoing that inhaling hydrogen–oxygen may promote the repair of lung injury and improve neurological symptoms for rehabilitation in post‐COVID‐19 (NCT05504460).

Last but not the least, efforts are needed to increase vaccination coverage, especially as prioritization population such as the elder, multicomorbidities, immunocompromised person, and children under the age of 5 years have not yet been vaccinated. To maintain nonspecific prevention strategies is necessary. The use of PHSM including physical distancing, cleaning hands, coughing to bent elbow or paper, and adequate ventilation in indoor environment and masks should be consistently and appropriately implemented.

## CONFLICT OF INTEREST

The authors report no conflicts of interest.

## ETHICS STATEMENT

None.

## AUTHOR CONTRIBUTIONS

Hou DN contributed in concept and design, manuscript preparation, and editing. Chen CC and Bi J did literature search and manuscript editing. She J designed the study and did manuscript preparation and editing. Song YL contributed in concept and design and reviewed this manuscript. The manuscript has been read and approved by all the authors. The requirements for authorship have been met. Each author believes that the manuscript represents honest work.

## Data Availability

Data sharing is not applicable to this article as no new data were created or analyzed in this study.
